# Choice of fluorophore affects dynamic DNA nanostructures

**DOI:** 10.1093/nar/gkab201

**Published:** 2021-03-30

**Authors:** Kevin Jahnke, Helmut Grubmüller, Maxim Igaev, Kerstin Göpfrich

**Affiliations:** Max Planck Institute for Medical Research, Biophysical Engineering Group, Jahnstraße 29, 69120 Heidelberg, Germany; Department of Physics and Astronomy, Heidelberg University, 69120 Heidelberg, Germany; Max Planck Institute for Biophysical Chemistry, Department of Theoretical and Computational Biophysics, Am Fassberg 11, 37077 Göttingen, Germany; Max Planck Institute for Biophysical Chemistry, Department of Theoretical and Computational Biophysics, Am Fassberg 11, 37077 Göttingen, Germany; Max Planck Institute for Medical Research, Biophysical Engineering Group, Jahnstraße 29, 69120 Heidelberg, Germany; Department of Physics and Astronomy, Heidelberg University, 69120 Heidelberg, Germany

## Abstract

The ability to dynamically remodel DNA origami structures or functional nanodevices is highly desired in the field of DNA nanotechnology. Concomitantly, the use of fluorophores to track and validate the dynamics of such DNA-based architectures is commonplace and often unavoidable. It is therefore crucial to be aware of the side effects of popular fluorophores, which are often exchanged without considering the potential impact on the system. Here, we show that the choice of fluorophore can strongly affect the reconfiguration of DNA nanostructures. To this end, we encapsulate a triple-stranded DNA (tsDNA) into water-in-oil compartments and functionalize their periphery with a single-stranded DNA handle (ssDNA). Thus, the tsDNA can bind and unbind from the periphery by reversible opening of the triplex and subsequent strand displacement. Using a combination of experiments, molecular dynamics (MD) simulations, and reaction-diffusion modelling, we demonstrate for 12 different fluorophore combinations that it is possible to alter or even inhibit the DNA nanostructure formation—without changing the DNA sequence. Besides its immediate importance for the design of pH-responsive switches and fluorophore labelling, our work presents a strategy to precisely tune the energy landscape of dynamic DNA nanodevices.

## INTRODUCTION

DNA nanotechnology has been highly successful in repurposing the iconic DNA double helix to create programmable molecular architectures. Once focused on static shapes, dynamic and stimuli-responsive DNA nanoscale devices are gaining a large surge of interest for various applications (1)—from sensors (2–4), biocomputing algorithms (5), and drug delivery systems (6,7) to programmable robotic modules (8,9) and functional components for synthetic cells (10–12). In a vast majority of such reconfigurable systems, dynamics are achieved using strand displacement reactions (13,14), flexible single-stranded hinges (15), stimuli-responsive DNA modifications (16,17) or sequence motifs (4,18). The ability to reversibly actuate artificial structures at the nanoscale is therefore at the core of dynamic DNA nanotechnology. Direct measurements of conformational changes in aqueous solutions are often conducted with Förster resonance energy transfer (FRET) or fluorescence microscopy (19–21). These methods can provide a readout of the overall conformational state of the structure, for example, open versus closed, or bound versus unbound. Hence, the use of fluorescent dyes is commonplace to validate and quantify the functionality of the DNA-based devices. Fluorophore-tagged DNA nanostructures have also been used as nanoscopic rulers for fluorescence microscopy (22) and to enable the acquisition of super-resolution images with DNA-PAINT (23). Factors like solubility, photostability and excitation/emission spectra usually play the decisive role in choosing a suitable dye, while potential side effects on the DNA conformation such as overstabilization of DNA duplexes (24) or specific fluorophore-DNA interactions (25) are not the main concern.

Here, we show that the choice of the fluorophore itself can alter the equilibrium conformation and even inhibit a desired dynamic response. We use a pH-responsive triple-stranded DNA motif (tsDNA) combined with a strand-displacement reaction to exemplify that the dynamics can be strongly influenced by the choice of the fluorophore. With all-atom molecular dynamics (MD) simulations, we show that fluorophore-dependent conformational dynamics of the single-stranded DNA (ssDNA) contribute to this observation. By releasing caged protons inside droplet-based confinement, we find that also the duplex dissociation is affected by the fluorophore. Using a reaction-diffusion model, we derive the apparent dissociation constant for 12 different experimentally tested fluorophore combinations. A profound knowledge about the effect that fluorophores and other chemical modifications have on the dynamics of a DNA-based system can be leveraged to realize the desired functionality.

## MATERIALS AND METHODS

### DNA sequence design

The DNA sequences were adapted from Green *et al.* (4). To enable self-assembly at the droplet-periphery, the ssDNA (termed ‘Regulator’ in Green *et al.*) was modified with a cholesterol-tag (sequence: 5′ Cy3/Alexa488/Cy5/-ACCAGACAATACCACACAATTTT-CholTEG 3′, HPLC purified). The tsDNA (termed ‘Sensor’ in Green *et al.*) contained the triple-stranded DNA motif as well as a stem loop complementary to the ssDNA. A fluorophore modification was added to its 5′ end (sequence: 5′ Cy5/Cy3/Atto488/Atto647-TTCTCTTCTCGTTTGCTCTTCTCTTGTGTGGTATTGTCTAAGAGAAGAG 3′, HPLC purified). Both DNA sequences were purchased from Biomers or Integrated DNA Technologies and dissolved in ultrapure water (Milli-Q) to exclude the impact of DNA storage buffer on the pH.

### Formation of DNA-containing water-in-oil droplets

For the formation of water-in-oil droplets, the DNA-containing aqueous phase was layered on top of the oil phase in a volumetric ratio of 1:3 within a microtube (Eppendorf). Droplet formation was induced by manual shaking for about 4 s as described earlier (26). For the oil-phase, 1.4 vol% of perflouro-polyether-polyethylene glycol (PFPE-PEG) block-copolymer fluorosurfactants (008-PEG-based fluorosurfactant, Ran Biotechnologies, Inc.) dissolved in HFE-7500 oil (DuPont) was used. The interfacially active surfactants stabilize the droplets. The aqueous phase was composed of 10 mM MgCl_2_ and 250 mM potassium phosphate buffer adjusted to pH values from 4.3 to 8.0. Cholesterol-tagged ssDNA and the tsDNA were added to the aqueous phase at concentrations of 1.66 and 1.25 μM, respectively, if not stated otherwise. ssDNA was added in excess to ensure that there are sufficient binding sites for the tsDNA. Other contents were encapsulated by adding them to the aqueous phase as described in text.

### Confocal fluorescence microscopy

For confocal microscopy, the DNA-containing droplets were sealed in a custom-built observation chamber and imaged 10 min after encapsulation using a confocal laser scanning microscope LSM 880 or LSM 800 (Carl Zeiss AG). The pinhole aperture was set to one Airy Unit and experiments were performed at room temperature. The images were acquired using a 20× objective (Plan-Apochromat 20×/0.8 M27, Carl Zeiss AG). Images were analysed and processed with ImageJ (NIH, brightness and contrast adjusted).

### Light-triggered proton release

To dynamically decrease the pH inside individual compartments, we co-encapsulated 40 mM NPE-caged sulfate (Santa Cruz Biotechnology), which undergoes photolysis upon illumination with light of the wavelength 405 nm and releases a proton. For the investigation of the detachment kinetics, 2 μM ssDNA and 1.5 μM tsDNA were mixed with 20 mM potassium phosphate buffer at pH 8 and 5 mM MgCl_2_. The use of the buffer ensures the same starting conditions and delays the acidification, which facilitates the imaging and analysis of the tsDNA fluorescence. After encapsulation, a subset of droplets was illuminated with 20$\%$ of the power of a 5 mW 405 nm laser diode while simultaneously recording the detachment of the Cy5-labelled tsDNA. The field of view, the laser intensity and all additional imaging conditions were kept the same.

### Image analysis

The tsDNA fluorescence inside the droplet and at the droplet periphery was analysed with a custom-written ImageJ macro (27). Droplets were identified and assigned a circular region of interest from which the droplet radius *r*_0_ was calculated. We first selected droplets of similar size in the range of 40−50 μm. Afterwards, we manually removed droplets which were in contact with each other and only analysed droplets without direct neighbours to avoid crosstalk of their fluorescent signals. Analysis of almost identical droplets reduces the variance in the measured quantities and allows for statistical robustness. Second, it unifies the acquired data such that mathematical modelling is possible. The intensity within the droplet center, *I*_in_, was defined as the mean intensity within a circle of radius *r*_in_ = 0.5*r*_0_. The intensity at the droplet periphery *I*_peri_ was quantified by measuring the maximum intensity along a line orthogonal to the droplet circumference. This analysis was repeated 20 times every 18° along the droplet circumference, and the mean value taken as *I*_peri_. Following the determination of the droplet intensities I_in_, they were plotted with Prism 8 (GraphPad) and fitted using a sigmoidal function of the form: $I_{\text{in}}=I_{\text{min}}+(I_{\text{min}}-I_{\text{max}})/(1+10^{-\alpha (\mathrm{pH}_{\text{turn}}-\mathrm{pH})})$, with α being the decay constant and pH_turn_ the p*K*_a_ and the turning point of the fit.

### Atomistic simulations of unlabelled ssDNA

To provide a realistic description of ssDNA dynamics both in the presence and in the absence of fluorescent dyes, we first performed a series of simulations for the dye-free ssDNA using the Parmbsc1 flavour (28) of the standard Amber 99SB force field (29) with CUFIX non-bonded corrections (30) and ion parameter corrections by Joung and Cheatham (31). We also used TIP3P as the water model in our simulations (32). The simulations were initiated from single-stranded helical structures built with Chimera (v. 1.14) (33). The starting structures were solvated in TIP3P water in a dodecahedron box with an edge length of 9.0 nm, yielding a system with ∼50 000 atoms. Ion concentrations were set to 250 mM NaCl and 10 mM MgCl_2_ to mimic the experimental conditions.

Subsequent MD simulations were performed with GROMACS 2019.6 (34). Lennard-Jones and short-range electrostatic interactions were calculated with a 1.0-nm cutoff, while long-range electrostatics was treated using particle-mesh Ewald summation (35) with a 0.12-nm grid spacing. Hydrogen bond lengths were constrained using the LINCS algorithm (36). Velocity rescaling (37) with a heat bath coupling constant of 1.0 ps was used to control the temperature for solute and solvent separately. Center-of-mass correction was applied to solute and solvent separately every 100 steps. Energy minimization was followed by a short equilibration for 1 ns in the NVT ensemble (*T* = 100 K) and with position restraints applied to the solute’s heavy atoms and a 1-fs integration time step. Next, the temperature was increased to *T* = 300 K, and the system was equilibrated for 5 ns (2-fs time step), while keeping the pressure at 1 atm using the Berendsen barostat (38) with a 1-ps coupling constant. The position restraints were then slowly released during 20 ns of equilibration in the NPT ensemble (*T* = 300 K, *p* = 1 atm, 2-fs time step) using the Parrinello-Rahman barostat (39). This initial equilibration step was followed by a total of 17 independent production runs, each being 5 μs long. The first ∼1  μs of the trajectories were discarded to exclude the initial relaxation towards the equilibrium state. Unless specified differently, all trajectory analyses were performed with Python (v. 2.7 available at https://www.python.org/), VMD (v. 1.9.2) (40) and Chimera (v. 1.14) (33).

### ssDNA simulations with fluorescent dyes covalently attached

Parameters and energy-minimized structures for common Alexa and Cy fluorescent dye families were derived from the AMBER-DYES library (41) that is compatible with most Amber force fields. Alexa488, Cy3 (water-soluble) and Cy5 (water-soluble) dyes were attached to the 5′ end of the ssDNA via a neutral lysine linker. To this end, the capping H5T atom of the 5′ nucleotide was removed, and the C atom of the linker backbone was bonded with the O5′ atom of the 5′ nucleotide. Since in the Amber formalism, the 5′ and 3′ nucleotides possess non-integer charges (−0.3e and −0.7e, respectively; unlike the regular nucleotides that have a charge of −1.0e), the resulting dye-ssDNA construct had a slightly non-integer charge. To account for this, the residual small charge was redistributed over the O5′, C5′, C4’, C3′, O4’, C1’ and C2’ atoms of the 5′ nucleotide (sugar backbone).

To accommodate the larger dye-ssDNA, the size of the simulation box was increased to 12 nm, yielding a system with about 120 000 atoms. All subsequent simulations were done under the same conditions as for the unlabelled ssDNA. For the dye-free simulations, multiple 6 μs production runs were performed and the first ∼1 μs were discarded as equilibration time. A summary of the simulated systems is given in Table 1.

**Table 1.** Summary of dye-free and dye-labelled ssDNA simulations

**Force field**	**System**	**Duration**
Parmbsc1 + TIP3P	No dye	17 × 5 μs
Parmbsc1 + TIP3P	Cy3	6 × 6 μs
Parmbsc1 + TIP3P	Cy5	6 × 6 μs
Parmbsc1 + TIP3P	Alexa 488	6 × 6 μs

### Determination of the apparent dissociation constant

The equilibrium distribution of ssDNA and tsDNA molecules in a droplet can be described mathematically using a reaction-diffusion system of equations in which the binding sites (ssDNA attached to the droplet periphery), and hence also the binding and dissociation reactions, are localized in a narrow volumetric layer near the spherical droplet surface (42,43). Briefly, if *S*_tot_ and *T*_tot_ are the total concentrations of ssDNA and tsDNA in the droplet, respectively, *T*_eq_ is the steady-state concentration of tsDNA in equilibrium, and *K*_D_ is the dissociation constant defining the ssDNA/tsDNA binding equilibrium, the ratio between the peripheral and inner intensity of tsDNA fluorescence can be expressed as:

(1) $$\begin{equation*} \frac{I_{\text{peri}}}{I_{\text{in}}} = 1 + \frac{S_{\text{tot}}}{T_{\text{eq}} + K_{\text{D}}}. \end{equation*}$$

Here, both *I*_in_ and *I*_peri_ are per-area intensities averaged over $\pi r^2_{\text{in}}$ and 2π*r*_0_ϵ, respectively, where ϵ is the apparent thickness of the reaction layer (determined from confocal images as described in the Supplementary Text S1). The steady-state concentration *T*_eq_,

(2) $$\begin{eqnarray*} T_{\rm eq} &=& \frac{1}{2}\left[-(3{\varepsilon}/{r_0}{S_{\rm tot}}+K_{\rm D}-{T_{\rm tot}})\right.\nonumber \\ && \left. + \sqrt{(3{\varepsilon}/{r_0}{S_{\rm tot}}+K_{\rm D}-{T_{\rm tot}})^2+4T_{\rm tot}K_{\rm D}} \right], \end{eqnarray*}$$

is obtained by simultaneously requiring that the ssDNA/tsDNA binding has attained equilibrium and that the number of tsDNA molecules in the droplet is conserved. The apparent *K*_D_ values presented in Figure 4 were determined using Equations (1) and (2), and the corresponding errors were calculated using basic error propagation rules and measured uncertainties of *I*_peri_, *I*_in_, *r*_0_ and ϵ. A detailed mathematical description of the model, estimation of ϵ, and error analysis are given in the Supplementary Texts S1 and S2.

### Radius of gyration distributions and estimations of confidence intervals

The gyration radii (*R*_g_) of ssDNA were calculated using the gmx gyrate tool included in the GROMACS package. The probability distributions *p*(*R*_g_) shown in Figure 2 were then computed by binning the corresponding data sets and normalizing the histograms. Confidence intervals for *p*(*R*_g_) were estimated using bootstrap analysis (44). To this end, we used the obtained distributions to bootstrap 10^6^ new random *R*_g_ samples (each consisting of 10^5^ data points) such that the newly generated data is distributed according to *p*(*R*_g_) and properly correlated with the autocorrelation time estimated from the original *R*_g_ trajectories.

## RESULTS

### Fluorophore modification influences pH response

**Figure 1.**
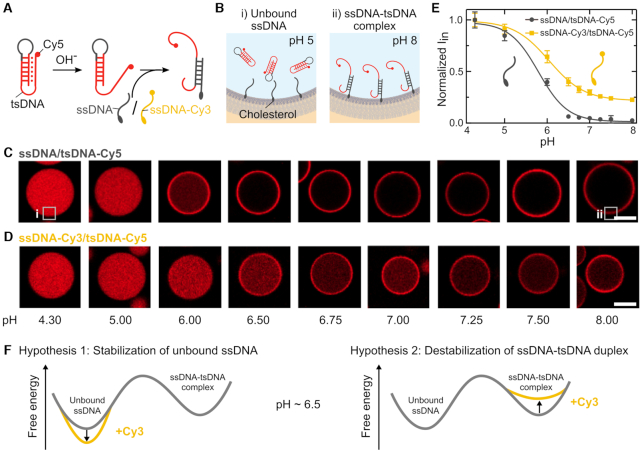
Fluorophore modification influences pH response. (**A**) Sketch of the pH responsive DNA motif. A Cy5-labelled triple-stranded DNA (tsDNA) opens up at basic pH, lowering the energy barrier for strand displacement and hence for complementary base pairing with a cholesterol-tagged single-stranded DNA (ssDNA). (**B**) This process can be monitored in water-in-oil droplets. The cholesterol-tagged ssDNA self-assembles at the droplet periphery, whereas cholesterol-free tsDNA remains homogeneously distributed within the droplet at acidic pH and attaches to the droplet periphery at higher pH values. (C, D) Representative confocal images of water-in-oil droplets containing Cy5-labelled tsDNA (red, λ_*ex*_ = 647 nm) and unlabelled ssDNA (**C**) or Cy3-labelled ssDNA (**D**) at different pH values. Attachment of the tsDNA is shifted to higher pH values if the ssDNA is labelled with Cy3. Scale bars: 20 μm. (**E**) Normalized steady-state fluorescence intensity of the Cy5-labelled tsDNA inside the droplet (periphery excluded) at different pH values for unlabeled (gray) and Cy3-labelled ssDNA (yellow). Error bars correspond to the standard deviation of the intensities of *n* = 20 droplets per condition. Solid lines represent sigmoidal fits revealing a turning point at pH 5.80 ± 0.09 and 6.05 ± 0.04, respectively. (**F**) Free energy profile illustrating potential hypotheses for the altered behaviour of the Cy3-tagged ssDNA compared to the unlabelled ssDNA.

We set out to test the impact of fluorophores on the dynamics of DNA nanostructures. For this purpose, we employed a popular triple-stranded DNA motif (tsDNA) (4) as an example. Its reversible pH-responsive actuation works as follows: At basic pH, the Hoogsten-interactions which stabilize the triple-stranded configuration are weaker than at acidic pH. Therefore, an increase in pH leads to unwrapping of the third strand which previously stabilized the duplex. This, in turn, lowers the energy barrier for a strand displacement reaction with a single-stranded DNA (ssDNA), which was designed to be complementary to the hairpin loop of the tsDNA. Thus, a stable DNA duplex can form between the tsDNA and the ssDNA (Figure 1A) (4). To monitor this process, we modified the ssDNA with a cholesterol-tag and encapsulated it together with the tsDNA into water-in-oil droplets. Upon encapsulation, the ssDNA self-assembled at the droplet periphery due to hydrophobic interactions between the cholesterol-tag and the droplet-stabilizing surfactants (11). Thereby, we obtained an attachment handle, which reversibly recruits the tsDNA to the periphery at basic pH (Figure 1B). In contrast to Förster Resonance Energy Transfer (FRET), which is commonly employed to monitor the pH dynamics (21), our system provides freedom regarding the choice of fluorophores—which is absolutely necessary for us to study the impact of different fluorophore combinations. We directly visualized tsDNA binding and investigated the impact of fluorophore modifications on the pH dynamics. At the same time, this system provides a strategic blueprint for the pH-sensitive recruitment of components to the membrane—an interesting function in itself, in particular concerning the bottom-up construction of synthetic cells (45).

Confocal imaging revealed that attachment of the tsDNA to the compartment periphery is shifted to higher pH values if the ssDNA carries a Cy3 compared to the unlabelled ssDNA (Figure 1C, D). The images show the equilibrated state (Supplementary Figure S1) and we confirmed that the shift is neither due to interactions of the Cy3 with the surfactant layer (Supplementary Figure S2) nor an artefact of the droplet size (Supplementary Figure S3). We quantified this effect by extracting the normalized intensity inside the droplets (*I*_in_, periphery excluded) from the confocal images (Figure 1E). Importantly, we could reproduce the sigmoidal pH response curve that was reported for FRET-based detection (46). The turning-point of the pH-sensitive ssDNA-tsDNA binding curve for unlabelled ssDNA was around 5.80 ± 0.09, whereas it shifted significantly to 6.05 ± 0.04 for the Cy3-modified ssDNA (2.53σ). Even at pH 8 not all tsDNA was bound to the droplet periphery for the Cy3-modified ssDNA. Importantly, we confirmed our results with polyacrylamide gel electrophoresis (PAGE) as an independent bulk measurement. Also with PAGE we find that the binding between ssDNA and tsDNA is enhanced if the ssDNA does not carry a fluorophore (Supplementary Figure S4). This confirms that our observations are not just an artifact of the measurement system itself, for example, due to unspecific interactions at the water-oil interface. Note that the p*K*_a_ of the DNA itself is far below the pH-range considered here and Cy3 is uncharged throughout the pH-range relevant for ssDNA-tsDNA binding with a p*K*_a_ of 7.8 (Supplementary Table S1).

While it is well known that the pH turning point can be shifted by changing the DNA sequence (46), it was not known that the same can be achieved by changing the fluorophore modification alone. This striking observation can be explained by either of the two following hypotheses as illustrated in Figure 1F: (i) a fluorophore modification on the ssDNA causes overstabilization of the free ssDNA state by making its equilibrium ensemble more compact and, therefore, less accessible for base paring. (ii) The fluorophore modification destabilizes the ssDNA-tsDNA complex, thereby raising the bound state in free energy (relative to the unbound one). First, we tested Hypothesis 1 with all-atom molecular dynamics (MD) simulations. Subsequently we examined Hypothesis 2 with experiments.

### MD simulations reveal reduction of ssDNA accessibility by fluorophore modification

**Figure 2.**
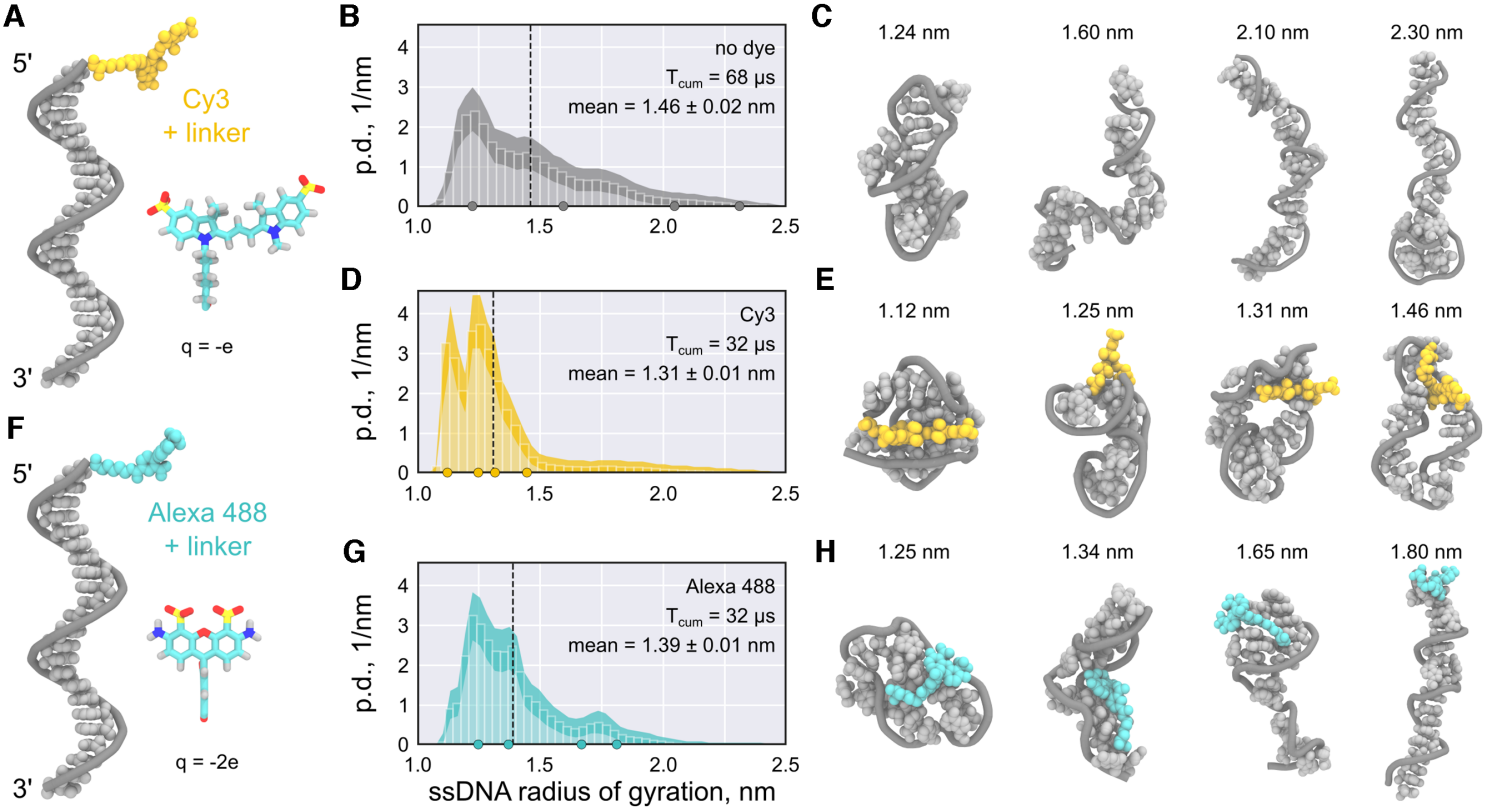
MD simulations suggest that fluorophore labelling can stabilize more compact ssDNA conformations. (**A, F**) Starting conformation of Cy3- (A, yellow) and Alexa488-labelled (F, turquoise) ssDNA (gray). The chemical structure of the fluorophore and its net charge are shown as an inset. (**B, D, G**) Probability density (p.d.) distributions of the gyration radius of ssDNA without dye (B), labelled with Cy3 (D), and labelled with Alexa488 (G). The shaded areas indicate the 95% confidence intervals estimated using bootstrapping (see Materials and Methods). The black dashed lines indicate the means of the distributions, *T*_cum_ the cumulative simulation time. (**C, E, H**) Representative structure snapshots of the unlabelled ssDNA (C), the Cy3-ssDNA (E) and the Alexa488-ssDNA (H). Positions of the selected snapshots within the corresponding distributions are marked with dots in the probability density distributions.

To test Hypothesis 1, we used all-atom MD simulations to probe the secondary structure of the Cy3-labelled ssDNA (Figure 2A) and compared it to the unlabelled ssDNA. First of all, the unlabelled ssDNA yielded a very broad probability density distributions for the radius of gyration (Figure 2B), which is a direct measure of the DNA’s compactness. The distributions for the unlabelled ssDNA show a significant fraction of extended structures, in which the DNA bases are accessible for complementary base pairing (see also representative snapshots in Figure 2C and Video S1). On the contrary, the Cy3-labelled ssDNA (Figure 2A) yielded a distinctively different probability density distribution for the radius of gyration (Figure 2D), which reflects a much lower propensity for extended conformations. The bases of the Cy3-labelled ssDNA were found to be wrapped around the fluorophore, most likely due to pi-pi stacking interactions between the ssDNA bases and the aromatic groups of Cy3 (Video S2). This entangled conformation renders the ssDNA less accessible for complementary base pairing. An overstabilization of the unbound ssDNA means a lower free energy of the ssDNA compared to the ssDNA–tsDNA complex. This would explain our experimental observations in line with Hypothesis 1. Note that Cy5-labelled ssDNA favored similarly compact conformations wrapped around the dye, which further indicates that the aromatic groups of Cy dyes tend to interact with ssDNA base pairs (Supplementary Figure S5, Video S3).

To test if weaker dye–ssDNA interactions would restore expanded conformations of the ssDNA in our simulations, we used an Alexa488 dye. We selected an Alexa dye (Figure 2F), because its chemical structure is considerably different compared to Cy3, which may render it less prone to base stacking interactions. Moreover, in contrast to the hydrophobic and highly planar Cy dyes, Alexa dyes are more hydrophilic due to their negative charge (Supplementary Table S1) (47) and smaller in size. We thus believe that a combination of electrostatic, steric and hydrophobicity-related effects could render Alexa dyes less prone to unspecific interactions with ssDNA, hence reducing the influence on the conformational ensemble of the ssDNA. We found that the mean radius of gyration for an Alexa488-modified ssDNA laid between that of the unmodified and the Cy3-modified ssDNA (Figure 2G). The MD snapshots show that the fully extended conformation, where the bases are accessible, was partially recovered (Figure 2H, Video S4), improving the accessibility of the strand for complementary base pairing.

Taken together, our simulations suggest that a single fluorophore modification on ssDNA can significantly change the DNA’s conformation. The more compact conformation of dye-labelled ssDNA effectively increases the free energy cost for expansion required for duplex formation with tsDNA. Thus, our simulations support Hypothesis 1. Importantly, the ssDNA sequence is random such that the observations can likely be generalized for a broad spectrum of DNA sequences.

### Dissociation kinetics show fluorophore dependence

**Figure 3.**
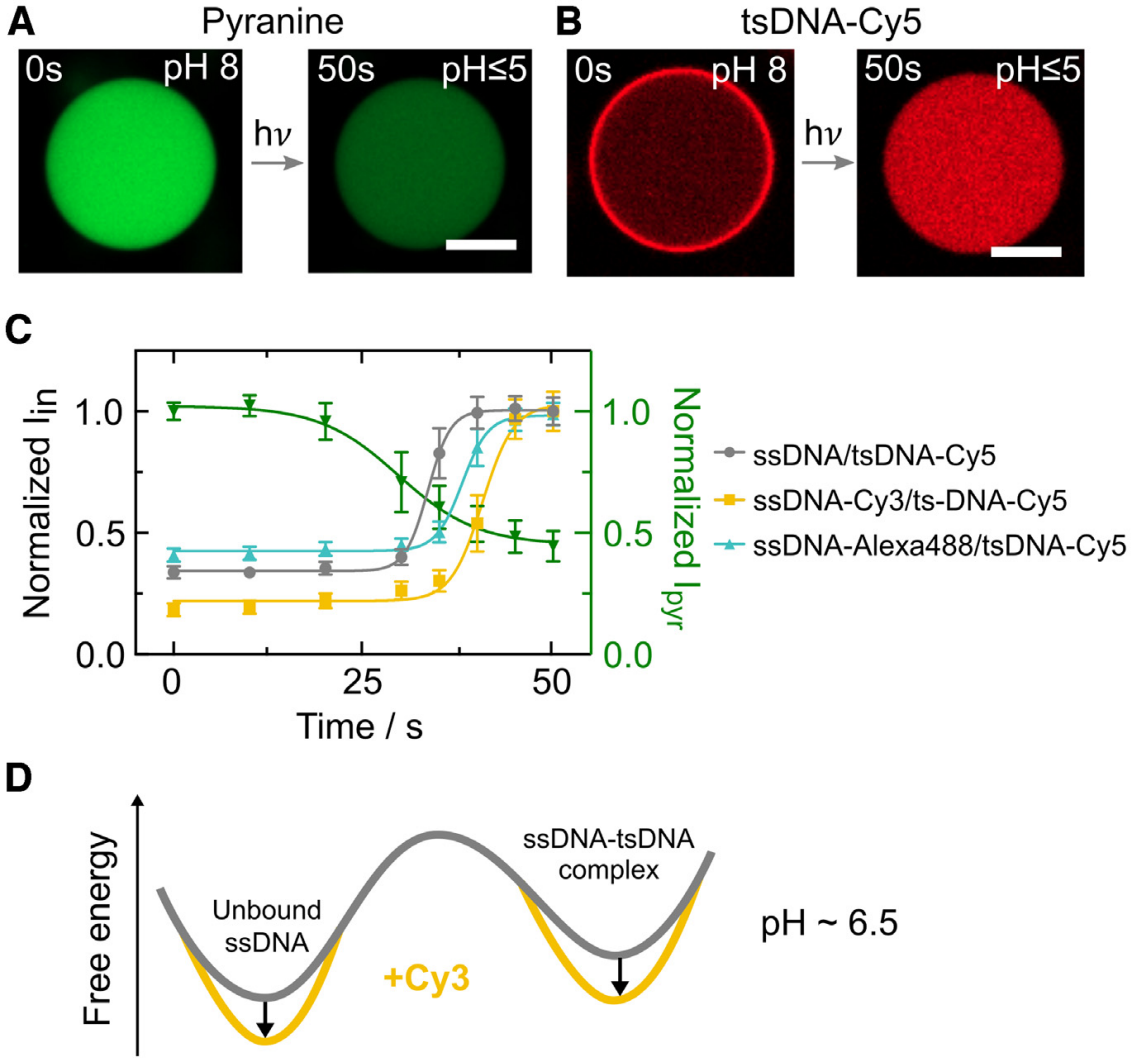
Light-mediated acidification of water-in-oil droplets reveals fluorophore-dependent duplex dissociation kinetics. (**A**) Confocal images of the pH-sensitive dye pyranine (50 μM, λ_ex_ = 488 nm, not coupled to DNA) encapsulated into water-in-oil droplets at pH 8. Light-triggered uncaging of NPE-caged sulfate (λ_ex_ = 405 nm) leads to proton release causing a rapid pH drop from 8 to under 5 within 50 s. The pH drop can be monitored as a decrease in pyranine fluorescence. (**B**) Representative confocal images of Cy5-labelled tsDNA (λ_ex_ = 647 nm) encapsulated together with cholesterol-tagged ssDNA into water-in-oil droplets at pH 8. During acidification, the tsDNA detaches from the droplet periphery as the triplex state is energetically favoured. Scale bars: 20 μm. (**C**) Normalized fluorescence intensity of the tsDNA inside the droplet (periphery excluded) over time for unlabelled, Cy3-labelled and Alexa488-labelled ssDNA as well as pyranine (right axis). Error bars correspond to the standard deviation of the intensities of *n* = 26 droplets for the ssDNA/tsDNA-Cy5 condition, *n* = 23 droplets for the ssDNA-Cy3/tsDNA-Cy5 condition, *n* = 24 droplets for the ssDNA-Alexa488/tsDNA-Cy5 condition and *n* = 5 droplets containing pyranine. Solid lines represent sigmoidal fits with turning points at 33.5 s ± 0.1 s (unmodified ssDNA), 40.7 s ± 0.5 s (ssDNA-Cy3) and 38.0 s ± 0.3 s (ssDNA-Alexa488). Note that the decay times *t*_d_ = 1/α are similar for all fluorophores 3.45 s ± 0.24 s (unmodified ssDNA), 4.76 s ± 1.13 s (ssDNA-Cy3) and 4.00 s ± 0.48 s (ssDNA–Alexa488). (**D**) Free energy profile illustrating our conclusion that both equilibrium states are stabilized by the presence of a dye on the ssDNA.

As a next step, we investigated the duplex dissociation process to see if the fluorophore modification affects the dissociation constant after duplex formation (Hypothesis 2). Since all-atom MD simulations cannot describe this reaction due to the limited timescales, we studied the detachment of the tsDNA from the compartment periphery experimentally. We implemented an approach where we achieved light-triggered release of protons in individual compartments—providing full spatio-temporal control over the acidification process. For this purpose, we used NPE-caged-sulfate, which breaks up into a sulfate and a proton upon photolysis (48). To prove that NPE-caged sulfate can be used to decrease the pH inside the droplets, we first encapsulated it together with the pH-sensitive dye pyranine at pH 8 and locally illuminated individual droplets with a 405 nm laser (Figure 3A). The pyranine emission upon 488 nm excitation decreased, confirming the successful pH decrease inside the droplets from initially pH 8 to under pH 5. The buffer kept the pH constant until its capacity is exceeded after ∼20 s. Then, the pH decreased until most of the NPE-caged sulfate underwent photolysis and hence the pH approached a constant value after ∼50 s. We used this dynamic light-mediated acidification mechanism to detach the tsDNA from the droplet periphery and to probe dissociation kinetics rather than the steady-state. At *t* = 0 s, the tsDNA was bound to the ssDNA at the droplet periphery (Figure 3B) and completely detached within 50 s of illumination. Upon detachment, the triplex conformation of the tsDNA was restored. In order to assess the detachment kinetics, we monitored the normalized tsDNA–Cy5 intensity for unmodified, Cy3-modified and Alexa488-modified ssDNA inside the droplet over time (Figure 3C). Following proton-release, the tsDNA detached from the ssDNA for all tested fluorophore modifications (Video S5). The decay times *t*_d_ = 1/α of the sigmoidal fits were comparable for all three fluorophore modifications, indicating similar detachment kinetics. However, detachment (i.e. duplex dissociation) occurred at different time points, hence at different pH values – again pointing towards an altered binding equilibrium. Detachment from the unlabelled ssDNA happened earlier (i.e. at higher pH) indicating that a fluorophore label is stabilizing the ssDNA-tsDNA complex. Taken together, the results obtained so far suggest that fluorophore modifications, in particular Cy-dyes, stabilize not only the unbound ssDNA (Hypothesis 1) but also the ssDNA-tsDNA duplex as illustrated in the free energy profile in Figure 3D. However, the stabilization of compact ssDNA conformations is likely stronger, which explains the observed shift of the pH switching point. This is effectively increasing the energy barrier for the dynamic switching of fluorophore-labelled DNA.

### Reaction-diffusion modelling reveals impact of fluorophores on apparent dissociation constant

**Figure 4.**
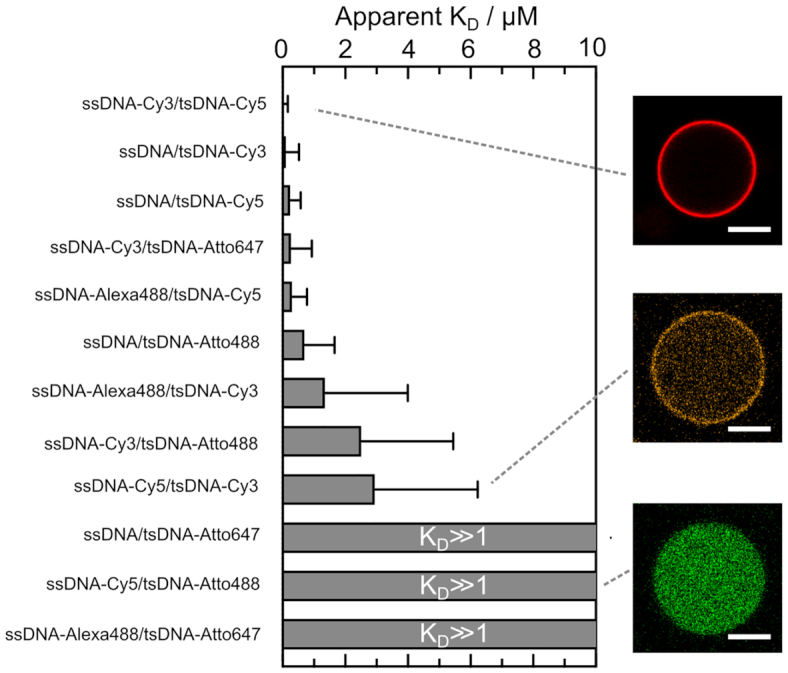
Histogram depicting the apparent dissociation constants *K*_D_ for 12 different ssDNA/tsDNA combinations at pH 8 with variable fluorophore modifications including Alexa488 (λ_ex_ = 488 nm), Atto488 (λ_*ex*_ = 488 nm), Cy3 (λ_*ex*_ = 561 nm), Cy5 (λ_ex_ = 647 nm) and Atto647 (λ_ex_ = 647 nm). Confocal images of three fluorophore combinations depicting strong (ssDNA–Cy3/tsDNA–Cy5), intermediate (ssDNA–Cy5/tsDNA–Cy3) and almost no binding to the droplet periphery (ssDNA–Cy5/tsDNA–Atto488). The apparent *K*_D_ is strongly influenced by fluorophore modifications on ssDNA and tsDNA up to the point of almost full inhibition of binding, which results in K_D_ ≫ 1. Error bars correspond to the standard deviation of *n* = 41 droplets for ssDNA/tsDNA–Cy5, *n* = 32 for ssDNA/tsDNA-Cy3, *n* = 11 for ssDNA/tsDNA–Atto488, *n* = 49 for ssDNA/tsDNA–Atto647, *n* = 73 for ssDNA–Cy3/tsDNA–Cy5, *n* = 29 for ssDNA–Cy3/tsDNA–Atto488, *n* = 67 for ssDNA–Cy3/tsDNA–Atto647, *n* = 27 for ssDNA–Cy5/tsDNA–Cy3, *n* = 31 for ssDNA–Cy5/tsDNA–Atto488, *n* = 72 for ssDNA-Alexa488/tsDNA-Cy5, *n* = 29 for ssDNA–Alexa488/tsDNA–Cy3 and *n* = 68 for ssDNA-Alexa488/tsDNA–Atto647.

Finally, having shown that a fluorophore modification on the ssDNA has a significant influence on the pH switching point, we now additionally tested the impact of fluorophore modifications on tsDNA. For this purpose, we investigated twelve different fluorophore combinations on ssDNA and tsDNA. To quantitatively compare the impact of different fluorophores, we developed an analytical model to derive the apparent dissociation equilibrium constant *K*_D_ = *k*_off_/*k*_on_ at a fixed pH for each individual fluorophore combination. For this purpose, we derived a reaction-diffusion model for spherical compartments (Text S2). It allowed us to determine the apparent dissociation constant *K*_D_ by extracting the droplet radius, the peripheral and the inner intensity of the tsDNA from confocal images with known total concentrations of DNA. We tested combinations of five different fluorophores, namely Cy3, Cy5, Alexa488, Atto488 and Atto647 as well as unlabelled ssDNA on the apparent *K*_D_ (Figure 4). Note that the use of an unlabelled tsDNA was not possible because it would inhibit the monitoring with confocal microscopy.

Remarkably, *K*_D_ varied dramatically for the different combinations. Most striking was the fact that binding is almost fully inhibited for certain fluorophore combinations, like ssDNA/tsDNA-Atto647, ssDNA–Cy5/tsDNA–Atto488 and ssDNA–Alexa488/tsDNA–Atto647 with *K*_D_ ≫ 1. On the other hand combinations like ssDNA–Cy3/ts–DNACy5, ssDNA/tsDNA–Cy3 and ssDNA/tsDNA–Cy5 bound very efficiently as expected at pH 8. As a general trend, we deduce that Cy-dyes on the tsDNA seemed to lead to a lower apparent *K*_D_ compared to Atto-dyes. Furthermore, it is surprising that the permutation of two Cy-dyes on ssDNA and tsDNA lead to a different apparent *K*_D_. While ssDNA-Cy3/tsDNA-Cy5 attached very efficiently, we obtained intermediate *K*_D_’s for ssDNA–Cy5/tsDNA–Cy3. This could be due to the fact that the larger Cy5 dye can interact with multiple bases and therefore stabilize the unbound ssDNA conformation more efficiently. Confirming our observations, the permutation of the tsDNA fluorophore influenced the pH hysteresis (Supplementary Figure S6) and the dynamic detachment in experiments using NPE-caged sulfate (Supplementary Figure S7). Moreover, if we move the fluorophore modification of the tsDNA from the 5′ end to the 3′ end, binding to the unlabelled ssDNA was enhanced (Supplementary Figure S8). This is likely due to the fact that the dye is placed further away from the stem loop, making the stem loop better accessible for complementary base-pairing with the ssDNA. In addition, a fluorophore modification on the 3′ end of the tsDNA may destabilize the triple-straned state, hence increasing the binding affinity for ssDNA. Indeed, due to the much smaller loop on that side, steric effects may come into play, which would equally contribute to the lower intensity inside the droplet for tsDNA-Cy5-3′.

Taking all these observations into account, we propose that not only a fluorophore modification on the ssDNA but also on the tsDNA affects the dynamics of pH-responsive DNA nanostructures up to a point that binding is inhibited. The choice of fluorescent dyes can thus be exploited to shape the energy landscape for dynamic DNA nanostructures and to shift the equilibrium towards the bound or the unbound state.

## DISCUSSION

One of the most exciting tasks in the field of DNA nanotechnology is the construction of dynamic molecular devices that can perform mechanical motion upon stimulation. The foundation for this work is an experimental readout, which is suitable to track dynamic reconfiguration in space and time. Fluorescence microscopy techniques, such as superresolution imaging or FRET, are ideally suited for *in situ* measurements on active DNA origami structures. The fluorophore is normally selected to match the optical setup rather than the DNA nanostructure itself and exchanged as required by the experiment. Here, we determined why the exchange of fluorophores on dynamic DNA nanostructures can lead to a considerably different experimental outcome. We used a popular pH-sensitive DNA motif combined with a strand displacement reaction as an example to show that the fluorophore alone can alter the steady-state and even completely inhibit the dynamics. Strand-displacement is one of the best understood and highly specific methods of actuating large DNA devices, but still has a large potential for improvement with respect to kinetics (49). Addressing this challenge, we find that fluorophores tend to stabilize the equilibrium states of the system with different effects on its dynamics, whereby Cy-dyes are more prone to inhibit dynamics compared to Atto-dyes. Beyond fluorophore labelling, DNA nanotechnology uses a myriad of other chemical modifications on the DNA, form reactive amine or thiol groups, hydrophobic tags, spacers, photocleavable groups or modifications for click chemistry (50). We anticipate that our observations are not limited to dye molecules – these other chemical modification would very likely have similar effects. It is thus generally possible to shape the energy landscape for dynamic reconfiguration as well as the equilibrium configuration without changing the DNA sequence.

Our results are directly relevant for various applications that capitalize on dynamic DNA systems, from bottom-up synthetic biology to biosensing and the the increasingly popular superresolution technique DNA-PAINT (23). Without doubt, the possibility to precisely shape energy landscapes for dynamic DNA nanostructures will lead to metastable DNA nanostructures and fully reversible DNA devices with unprecedented complexity – mimicking the intricate workings of natural nanomachines.

## DATA AVAILABILITY

The experimental data sets were appended to this submission. The MD trajectories, encompassing several TB of data, are available from the authors upon request.

## Supplementary Material

gkab201_Supplemental_Files
